# Long-term outcomes with emicizumab in hemophilia A without inhibitors: results from the HAVEN 3 and 4 studies

**DOI:** 10.1016/j.rpth.2024.102364

**Published:** 2024-03-01

**Authors:** Johnny Mahlangu, Víctor Jiménez-Yuste, Giuliana Ventriglia, Markus Niggli, Simona Barlera, Cédric Hermans, Michaela Lehle, Pratima Chowdary, Lyle Jew, Jerzy Windyga, Laurent Frenzel, Christophe Schmitt, Giancarlo Castaman, Steven W. Pipe

**Affiliations:** 1 Faculty of Health Sciences, University of the Witwatersrand, National Health Laboratory Service, Johannesburg, South Africa; 2 Jefe de Servicio de Hematología, La Paz University Hospital-IdiPaz, Autónoma University, Madrid, Spain; 3 Oncology and Hematology Product Development, F. Hoffmann-La Roche Ltd, Basel, Switzerland; 4 Product Development Data Sciences, F. Hoffmann-La Roche Ltd, Basel, Switzerland; 5 Department of Biometrics, Parexel International, Milan, Italy; 6 Haemostasis and Thrombosis Unit, Division of Haematology, Cliniques Universitaires Saint-Luc, Catholic University of Louvain, Brussels, Belgium; 7 Katharine Dormandy Haemophilia and Thrombosis Unit, Royal Free London, London, United Kingdom; 8 Product Development Safety, Genentech, Inc. South San Francisco, California, USA; 9 Department of Hemostasis Disorders and Internal Medicine, Laboratory of Hemostasis and Metabolic Diseases, Institute of Hematology and Transfusion Medicine, Warsaw, Poland; 10 Department of Hematology, Necker-Enfants Malades Hospital, Paris, France; 11 Department of Clinical Pharmacology, F. Hoffmann-La Roche Ltd, Basel, Switzerland; 12 Center for Bleeding Disorders and Coagulation, Careggi University Hospital, Florence, Italy; 13 Departments of Pediatrics and Pathology, University of Michigan, Ann Arbor, Michigan, USA

**Keywords:** efficacy, emicizumab, hemophilia A, prophylaxis, safety

## Abstract

**Background:**

Emicizumab, a bispecific monoclonal antibody, bridges activated factor (F) IX and FX, mimicking the function of missing or deficient activated FVIII in people with hemophilia A (HA).

**Objectives:**

To evaluate the long-term efficacy and safety of emicizumab prophylaxis in people with HA without FVIII inhibitors in the HAVEN 3 and 4 studies.

**Methods:**

HAVEN 3 and 4 were phase 3 open-label studies. Participants received emicizumab maintenance doses of 1.5 mg/kg every week or 3 mg/kg every 2 weeks (HAVEN 3), or 6 mg/kg every 4 weeks (HAVEN 4). Long-term efficacy and safety were assessed.

**Results:**

A total of 151 and 40 individuals without FVIII inhibitors received emicizumab in HAVEN 3 and 4, respectively. At the last patient, last visit dates (May 12, 2022 [HAVEN 3] and June 29, 2022 [HAVEN 4]), the median (range) duration of emicizumab exposure across the 2 studies was 248.1 (6.1-287.1*)* weeks. The mean (95% CI) annualized bleed rate for treated bleeds was 2.0 (0.23-7.15) for weeks 1 to 24, decreasing to 0.9 (0.01-5.28) by weeks 217 to 240. Overall, 188 (98.4%) participants experienced ≥1 adverse event (AE), with 185 treatment-related AEs in 71 (37.2%) participants. Forty-four (23.0%) participants reported a serious AE. Two thromboembolic events were reported, which were deemed unrelated to emicizumab by the investigator. No thrombotic microangiopathies were reported.

**Conclusion:**

With nearly 5 years of emicizumab exposure across the HAVEN 3 and 4 studies in people with HA without inhibitors, these data indicate continued bleed control with no new safety signals observed during long-term follow-up.

## Essentials

• Data on long-term use of emicizumab in people with hemophilia A without inhibitors are limited.
• Emicizumab prophylaxis in people with hemophilia A without inhibitors was evaluated.
• Annualized bleed rates were low, with no new safety signals during long-term follow-up.
• Five years of follow-up data showed a favorable benefit-risk profile of emicizumab prophylaxis.

## Graphical abstract

**Figure:**
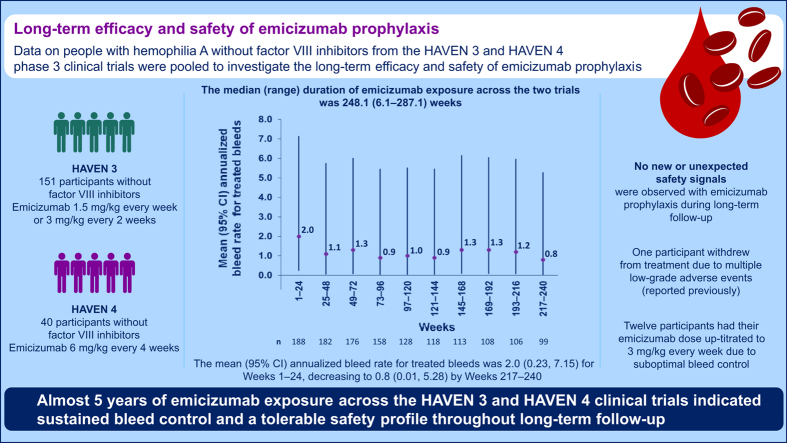

## 1 Introduction

Hemophilia A (HA) is a rare, inherited bleeding disorder that is characterized by a deficiency in coagulation factor (F) VIII, leading to recurring bleeding episodes [1]. Bleeding into joints is one of the most common symptoms experienced by people with severe HA, with recurrent episodes resulting in hemarthrosis, arthropathy, and chronic synovitis [1,2].

Historically, prophylactic factor replacement therapy, administered intravenously multiple times per week, has been the standard of care for managing bleeds and preventing joint damage in people with HA [1].

Emicizumab is the first non-factor therapy approved for HA [[3], [4], [5]]. It is a bispecific monoclonal antibody that bridges activated FIX and FX, mimicking the function of missing or deficient activated FVIII in people with HA. It can be administered subcutaneously every week (QW), every 2 weeks (Q2W), or every 4 weeks (Q4W) as prophylaxis [4,5]. In HAVEN 3 (NCT02847637), emicizumab was given at a maintenance dose of 1.5 mg/kg QW or 3 mg/kg Q2W and was shown to be efficacious and well tolerated in individuals without FVIII inhibitors in the primary analysis [6]. HAVEN 4 (NCT03020160) demonstrated the efficacy and tolerability of an emicizumab maintenance dose of 6 mg/kg given Q4W in people with HA with or without FVIII inhibitors [7].

Previous studies evaluating the long-term safety and efficacy of emicizumab prophylaxis have been conducted; however, data on the long-term outcomes of emicizumab prophylaxis, specifically in individuals without FVIII inhibitors, are limited. Here, we describe the data on people with HA without FVIII inhibitors from the long-term follow-up of the HAVEN 3 and 4 studies.

## 2 Methods

### 2.1 Study design and participants

This HAVEN 3 and 4 study analysis evaluates long-term outcomes of emicizumab prophylaxis in people with HA without FVIII inhibitors at study closure (last patient, last visit [LPLV]).

**Figure 1:**
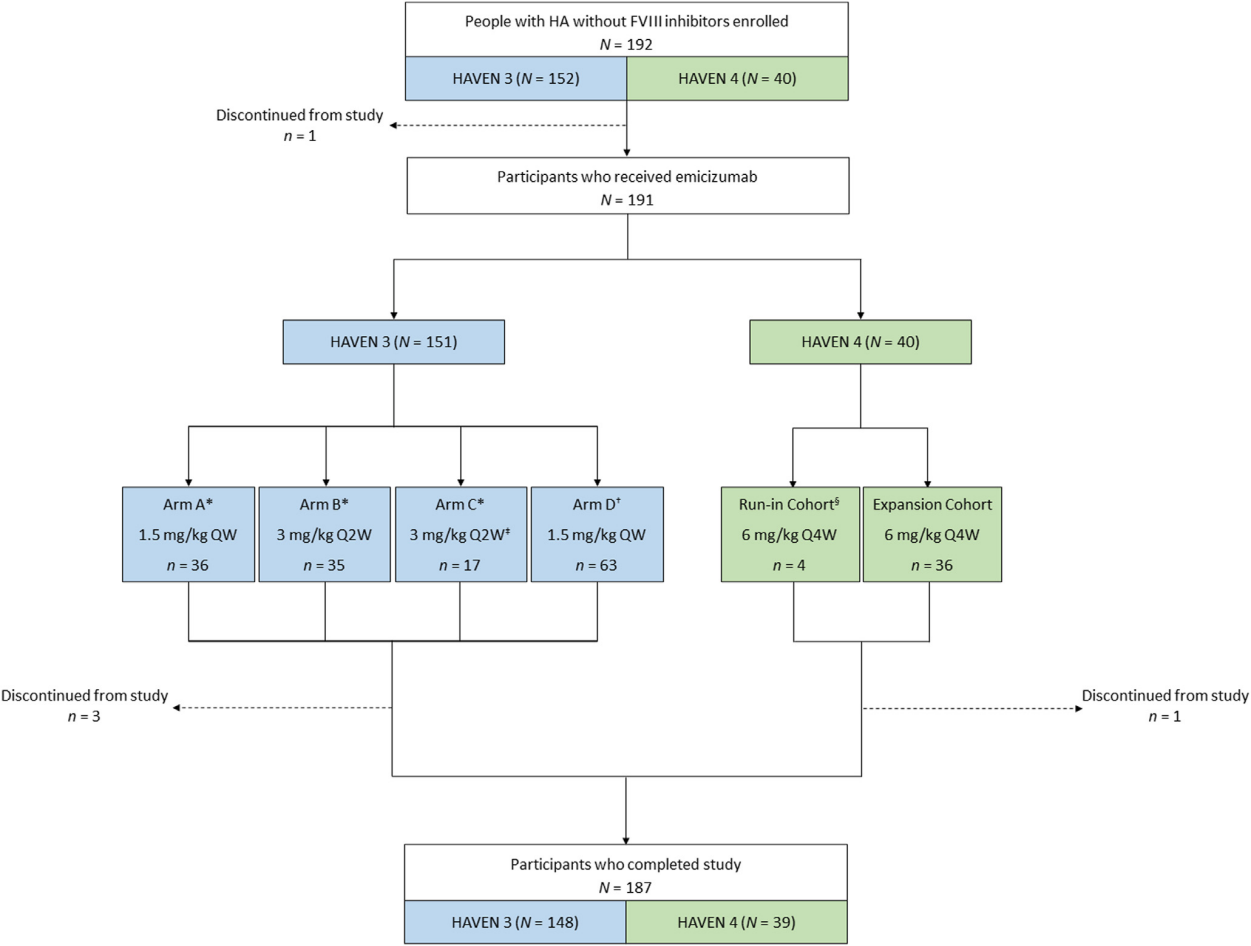
Participant flowchart. ∗Participants received episodic factor VIII prior to study entry; ^†^Participants received prophylactic FVIII prior to study entry; ^‡^Participants received no prophylaxis until week 24, after which they were given a loading dose of emicizumab 3 mg/kg QW, followed by a maintenance dose of 3 mg/kg Q2W; ^§^No loading dose period was included for this cohort. F, factor; HA, hemophilia A; QW, once weekly; Q2W, once every 2 weeks; Q4W, once every 4 weeks.

HAVEN 3 was a phase 3, open-label, multicenter, randomized trial. Eligible participants were males and females, 12 years of age or older, with severe HA (FVIII activity < 1 IU/dL), without FVIII inhibitors, who were receiving treatment with episodic or prophylactic FVIII [6]. Participants who had previously been receiving episodic treatment with FVIII were randomized to receive emicizumab or no prophylaxis (Figure 1). Participants in the emicizumab arms received a loading dose of 3 mg/kg QW for 4 weeks, followed by a maintenance dose of either 1.5 mg/kg QW (arm A) or 3 mg/kg Q2W (arm B). Arm C was the control arm, where participants received no prophylaxis for up to 24 weeks. On completion of 24 weeks, participants in arm C received a loading dose of emicizumab 3 mg/kg QW for 4 weeks, followed by a maintenance dose of 3 mg/kg Q2W. Participants who had previously been receiving prophylactic treatment with FVIII prior to the study formed arm D and were treated with a loading dose of emicizumab 3 mg/kg QW for 4 weeks, followed by 1.5 mg/kg QW thereafter.

HAVEN 4 was a phase 3, open-label, multicenter, nonrandomized study with a 2-stage study design (a run-in cohort and an expansion cohort) [7]. Eligible participants were males and females, 12 years of age or older, with severe HA (FVIII activity < 1 IU/dL) or HA with FVIII inhibitors, who were receiving treatment with episodic or prophylactic FVIII or bypassing agents. In both the run-in and expansion cohorts, participants received emicizumab 6 mg/kg Q4W (Figure 1). For the expansion cohort only, this was preceded by a loading dose of 3 mg/kg QW for 4 weeks. Only the participants without FVIII inhibitors, from both the run-in and expansion cohorts, were included in the present long-term analysis.

The complete inclusion and exclusion criteria for HAVEN 3 and 4 have been previously reported [6,7].

For both HAVEN 3 and 4, from 24 weeks after study enrollment, participants could have their emicizumab dose up-titrated to 3 mg/kg QW if they had suboptimal bleed control. This was defined as ≥2 spontaneous bleeds, verified (eg, with diagnostic imaging, physical examination, or a photograph) and considered clinically significant by a physician, during the previous 24 weeks, both of which occurred after the end of the loading dose period [8]. For the HAVEN 4 study only, subsequent dose down-titration was also allowed at the discretion of the treating physician. In addition, during the extension phase of both studies, participants were allowed the opportunity to switch to any of the approved emicizumab dosing regimens (1.5 mg/kg QW, 3 mg/kg Q2W, or 6 mg/kg Q4W), according to personal preference.

These studies were conducted in accordance with the Declaration of Helsinki, applicable local regulations, the Council for International Organizations of Medical Sciences International Ethical Guidelines, and Good Clinical Practice guidelines and local laws. The protocols were approved by the Institutional Review Board/Ethics Committee at each participating site. Written informed consent was obtained from participants before commencement of the study.

### 2.2 Study endpoints

The primary efficacy endpoint in both HAVEN 3 and 4 was annualized bleed rate (ABR) for treated bleeds, which were defined as a bleeds followed by treatment with a hemostatic agent.

Secondary efficacy endpoints included ABRs for treated joint bleeds; treated target joint bleeds, where target joints are defined as joints with ≥3 bleeds occurring in the same joint over 24 weeks [9]; treated spontaneous bleeds; and all bleeds. Participants reported bleeds via a Bleed and Medication Questionnaire (BMQ). FVIII usage was assessed through annualized infusion rates and annualized consumption (Units/kg). Target joint resolution was also evaluated, with target joints considered resolved if there were ≤2 bleeds into that joint in a 12-month period [9].

The key safety endpoints included the incidence and severity of adverse events (AEs); serious AEs (SAEs); AEs of special interest, which included thromboembolic events (TEs) and thrombotic microangiopathies; AEs leading to drug discontinuation/modification; incidence of antidrug antibodies (ADAs); and development of *de novo* FVIII inhibitors.

The development of ADAs was assessed in plasma samples using a validated enzyme-linked immunosorbent assay (ELISA) [10]. The neutralizing capacity of ADAs was evaluated using a FVIII chromogenic assay measuring emicizumab activity.

### 2.3 Statistical analysis

Descriptive statistics are presented. ABRs are given as medians with ranges and means with 95% CIs. Model-based ABRs were additionally derived, using a negative binomial regression model.

ABRs were calculated from data pooled across HAVEN 3 and 4 in discrete, consecutive 24-week periods. For each 24-week interval, only participants exposed to emicizumab during the 24-week period were included in the ABR calculations. Each 24-week period required available data from at least 10 participants to calculate the ABR. As a supportive analysis, mean and median ABRs were also calculated for discrete consecutive 52-week time intervals. Model-based ABRs were used for variable time periods (ie, when considering bleeds across the entire study period) as this method considers the variations in exposure times between participants. Exact Poisson and Clopper–Pearson methods were used to determine CIs.

Annualized infusion rates and annualized consumption of FVIII usage were calculated by dividing the number of infusions or quantity of FVIII, respectively, administered within a time period by the duration of that period in days, multiplied by 365.25. Participants who received FVIII prophylaxis before enrollment in HAVEN 3 (arm D) and HAVEN 4 were permitted to continue it for 1 week after the start of emicizumab; therefore, FVIII consumption data for the first week of emicizumab prophylaxis were excluded for those participants. For participants randomized to the control arm in HAVEN 3 (arm C), only the data after initiation of emicizumab prophylaxis were included in this analysis.

As assessment of target joint resolution required analysis of 12 consecutive months, participants were only eligible for this analysis if they had received ≥12 months of emicizumab.

The efficacy period for each individual started on the first day of emicizumab prophylaxis and ended on the date of clinical cutoff (LPLV), the day of study withdrawal, or the day before dose up-titration/down-titration, whichever occurred first. This period includes the time before or after switching a dosing frequency, where applicable. The safety period for each individual started on the first day of emicizumab prophylaxis and ended on the date of clinical cutoff (LPLV) or the day of study withdrawal, whichever occurred first (including periods with a change in dosing frequency or up- or down-titration dose).

## 3 Results

### 3.1 Participant demographics and baseline characteristics

As of the LPLV dates (May 12, 2022, for HAVEN 3 and June 29, 2022, for HAVEN 4), a total of 152 and 40 people with HA without FVIII inhibitors had been enrolled in HAVEN 3 and 4, respectively (Table 1 and Figure 1). Four of the 40 participants without FVIII inhibitors enrolled in HAVEN 4 were from the run-in cohort, and 36 were from the expansion cohort. There were 191 participants across the 2 studies who received emicizumab treatment. One participant enrolled in arm C of HAVEN 3 was lost to follow-up before completing 24 weeks of the study, and therefore was not exposed to emicizumab and is not included in this analysis.

**Table 1:** Participant baseline characteristics.

Characteristic	HAVEN 3 (*N* = 151)	HAVEN 4 (*N* = 40)	Total (*N* = 191)
Age (y), median (minimum-maximum)	38.0 (13-77)	40.0 (14-66)	38.0 (13-77)
Age group, *n* (%)			
<18 y	8 (5.3)	3 (7.5)	11 (5.8)
≥18 y	143 (94.7)	37 (92.5)	180 (94.2)
Race, *n* (%)			
American Indian or Alaska Native	0	0	0
Asian	31 (20.5)	7 (17.5)	38 (19.9)
Black or African American	8 (5.3)	1 (2.5)	9 (4.7)
Native Hawaiian or other Pacific Islander	1 (0.7)	0	1 (0.5)
White	102 (67.5)	31 (77.5)	133 (69.6)
Multiple	0	0	0
Unknown	9 (6.0)	1 (2.5)	10 (5.2)
Record of ≥1 CV risk factor^a^, *n* (%)	52 (34.4)	15 (37.5)	67 (35.1)
Treatment regimen at baseline, *n* (%)			
Episodic	88 (58.3)	13 (32.5)	101 (52.9)
Prophylactic	63 (41.7)	27 (67.5)	90 (47.1)
No. of bleeds (24 weeks prior to study)			
Mean (SD)	13.1 (16.2)	9.2 (15.7)	12.2 (16.2)
Median (minimum-maximum)	9.0 (0-128)	5.0 (0-90)	8.0 (0-128)
IQR	3.0-17.0	2.0-10.5	3.0-16.0
Target joints (prior to study)			
No. of participants, *n* (%)	97 (64.2)	26 (65.0)	123 (64.4)
No. of target joints, *n*	238	68	306
Mean (SD)	1.7 (1.6)	1.8 (1.9)	1.7 (1.7)
Median (minimum-maximum)	1.0 (0-6)	1.0 (0-8)	1.0 (0-8)
IQR	0.0-3.0	0.0-3.0	0.0-3.0

CV, cardiovascular; IQR, interquartile range; SD, standard deviation.

a Defined as a past medical history of CV disease or prior stroke; current evidence of hypertension, diabetes, hyperlipidemia, or obesity (defined as a body mass index ≥30 kg/m^2^).

The median (range) observation time was 262.3 (17.3-288.3) weeks for HAVEN 3 and 251.9 (71.9-276.3) weeks for HAVEN 4. The median (range) duration of exposure across the 2 studies was 248.1 (6.1-287.1) weeks, with a total of 729.3 participant-years of exposure. A total of 5 participants from HAVEN 3 and 4 discontinued their study. These included 4 participants in HAVEN 3, 1 each in arms A and C who were lost to follow-up, and 1 each in arms A and B who withdrew their consent to participate in the study. The participant in arm B withdrew their consent to participate due to multiple low-grade AEs. Further, 1 participant in the HAVEN 4 run-in cohort discontinued from the study due to self-withdrawal. The decline in the number of study participants from week 1 to week 240 was predominantly due to study completion, with 187 (97.9%) participants completing their respective study. Of these, 38 (20.3%) joined a posttrial access program, while the remaining 149 (79.7%) switched to commercial emicizumab.

In both HAVEN 3 and 4, all study participants were male. The total number of participants included in this analysis who were receiving episodic treatment at baseline was 101 (52.9%), while 90 (47.1%) received prior prophylactic treatment. Participants (*N* = 191) had a median (range) of 8.0 (0-128) bleeds (treated or untreated) within the 24 weeks before study entry. A total of 48 participants in HAVEN 3 who were receiving prophylactic FVIII treatment before the study (arm D) were also part of a previous noninterventional study [11]. On completion of the noninterventional study, participants could transfer to the corresponding HAVEN study, ie, individuals without inhibitors could enroll in HAVEN 3. The model-based ABR (95% CI) for treated bleeds for these 48 participants was 4.8 (3.22-7.09) before their enrollment in HAVEN 3 [11] (Supplementary Table S1).

In the overall population, 123 (64.4%) participants had at least 1 target joint at baseline. The median (range) number of target joints was 1.0 (0-8).

### 3.2 Efficacy

Compliance with bleed reporting via the BMQ was 93.3% in HAVEN 3 and 91.1% in HAVEN 4, with an overall compliance rate of 92.9% across the 2 studies.

**Figure 2:**
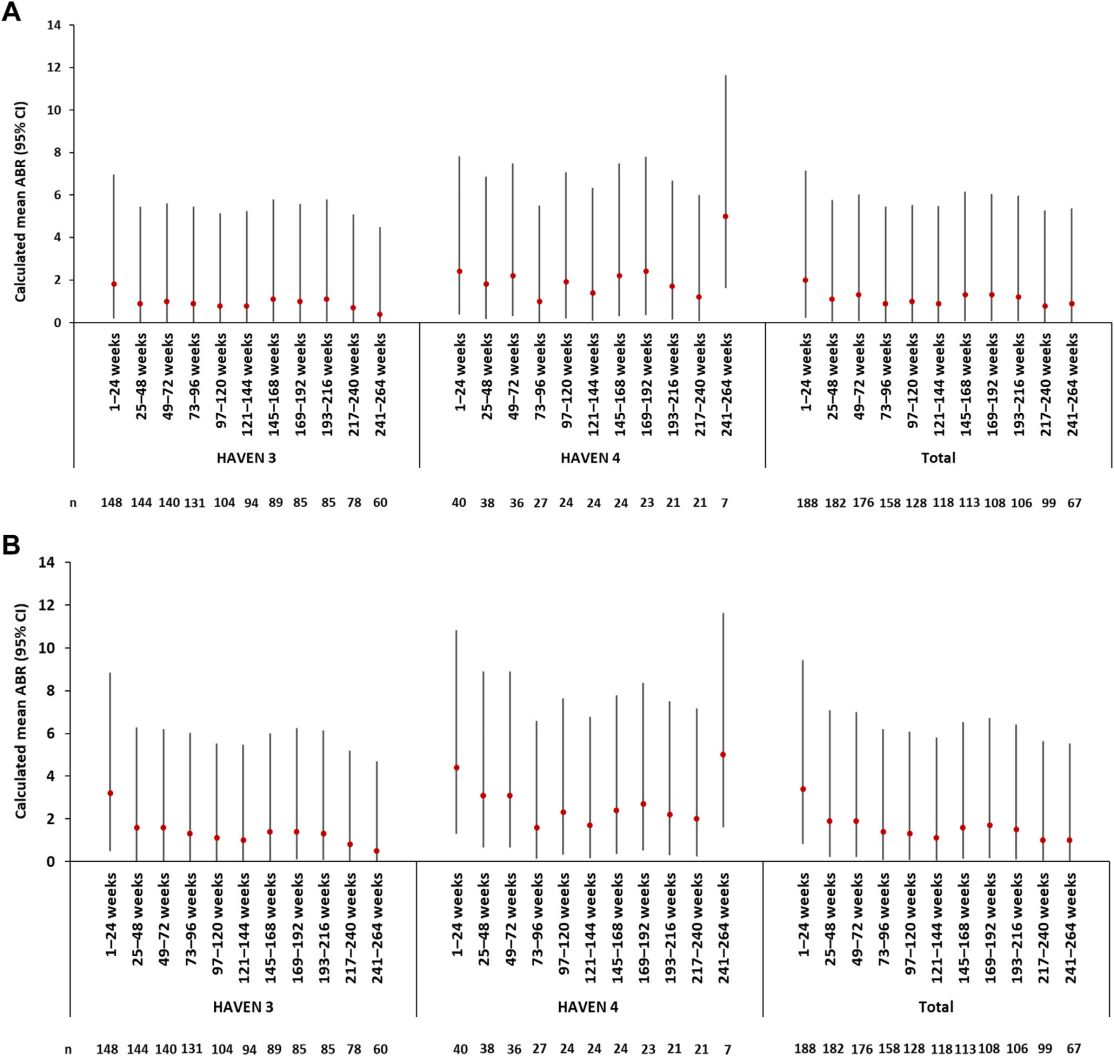
Mean annualized bleeding rates (ABRs) for (A) treated bleeds and (B) all bleeds over 24-week intervals.

The calculated mean (95% CI) ABR for treated bleeds for the pooled population of the 2 studies was 2.0 (0.23-7.15) for weeks 1 to 24 (*n* = 188) (Figure 2A and Supplementary Table S2). This decreased to 0.8 (0.01-5.28) by weeks 217 to 240 (*n* = 99), the latest timepoint at which more than 50% of participants were still in the overall studies. For all bleeds, the mean (95% CI) ABR decreased from 3.4 (0.82-9.43) at weeks 1 to 24 (*n* = 188) to 1.0 (0.03-5.63) at weeks 217 to 240 (*n* = 99; Figure 2B). ABRs for treated spontaneous bleeds, treated joint bleeds, and treated target joint bleeds also decreased from the first 24-week period onwards (Supplementary Table S2). Calculated mean ABRs were also assessed over 52-week intervals, with all bleed types showing an overall decrease from weeks 1 to 52 to weeks 209 to 260 (Supplementary Table S3). For treated bleeds for the overall population, the mean (95% CI) ABR decreased from 1.4 (0.08-6.22) at weeks 1 to 52 to 0.8 (0.01-5.19) at weeks 209 to 260 (Supplementary Figure S1).

**Figure 3:**
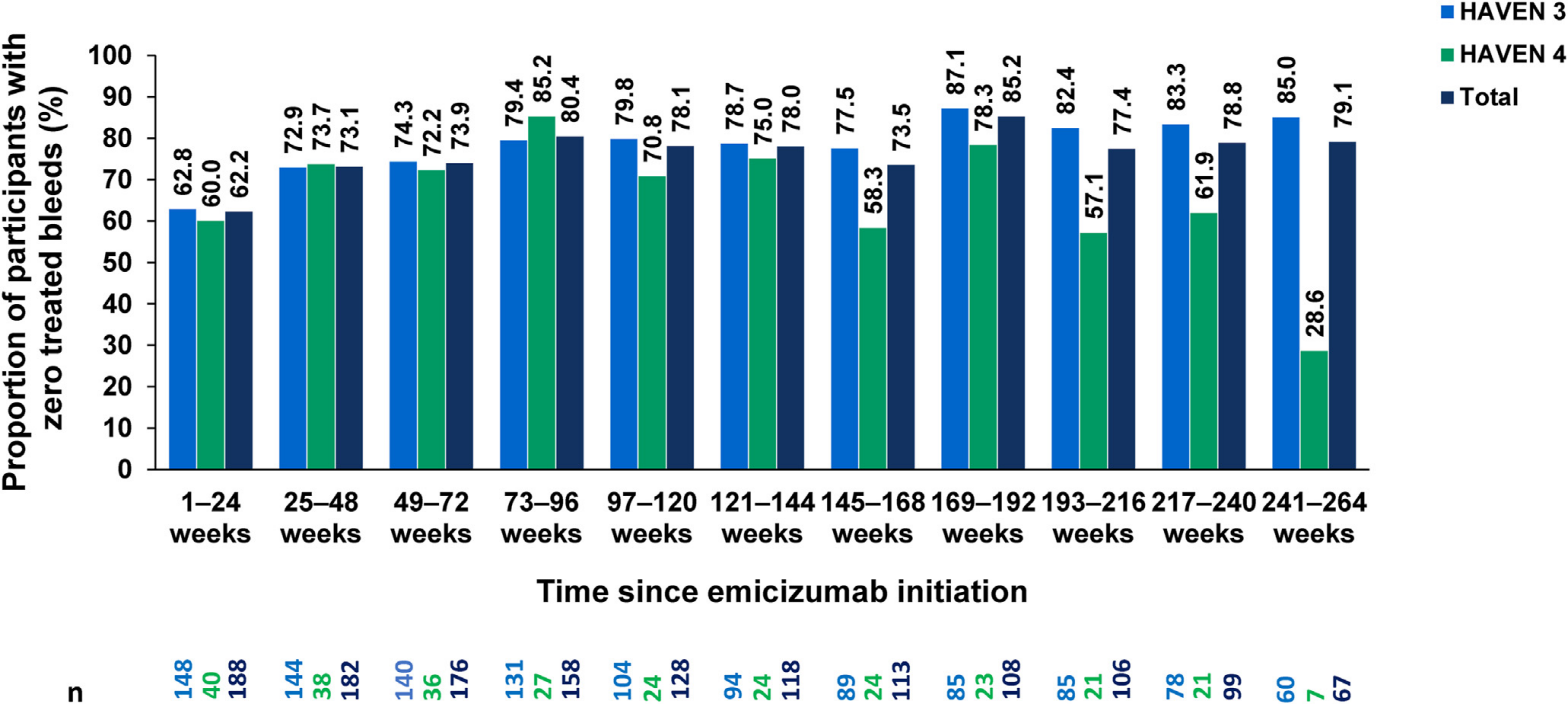
Proportion of participants with zero treated bleeds over 24-week intervals.

The model-based ABRs (95% CI) for the overall population were as follows: 1.4 (1.10-1.74) for treated bleeds, 0.9 (0.66-1.15) for treated joint bleeds, 0.5 (0.35-0.71) for treated target joint bleeds, 0.4 (0.33-0.60) for treated spontaneous bleeds, and 2.1 (1.73-2.54) for all bleeds (Table 2). When evaluated over successive 24-week periods, the proportion of participants with zero treated bleeds increased from 62.2% (117/188) in weeks 1 to 24 to 78.8% (78/99) in weeks 217 to 240 (Figure 3). The decline in the total number of study participants evaluated in weeks 217 to 240 was predominantly due to study completion. When evaluated over 52-week periods, the proportion of participants with zero treated bleeds increased from 50.5% (92/182) in weeks 1 to 52 to 70.5% (55/78) in weeks 209 to 260 (Supplementary Figure S2).

**Table 2:** Annualized bleeding rates for the different types of bleeds following emicizumab initiation until clinical cutoff^a^.

		Treated bleeds	Treated joint bleeds	Treated target joint bleeds	Treated spontaneous bleeds	All bleeds
HAVEN 3 (*N* = 151)	Model-based^b^ ABR (95% CI)	1.2 (0.92-1.57)	0.8 (0.55-1.05)	0.5 (0.30-0.68)	0.4 (0.29-0.59)	1.8 (1.47-2.30)
	Mean ABR (95% CI)	1.3 (0.07-6.08)	0.8 (0.01-5.28)	0.5 (0.00-4.66)	0.5 (0.00-4.68)	2.0 (0.24-7.19)
	Median ABR (IQR)	0.4 (0.00-1.15)	0.2 (0.00-0.72)	0.0 (0.00-0.38)	0.0 (0.00-0.41)	1.0 (0.19-2.37)
	Minimum-maximum	0.00-18.87	0.00-13.48	0.00-12.89	0.00-8.64	0.00-25.90
HAVEN 4 (*N* = 40)	Model-based^b^ ABR (95% CI)	2.1 (1.37-3.13)	1.3 (0.77-2.18)	0.7 (0.32-1.36)	0.5 (0.30-0.98)	3.0 (2.13-4.37)
	Mean ABR (95% CI)	2.2 (0.30-7.50)	1.4 (0.08-6.19)	0.7 (0.00-4.98)	0.7 (0.00-4.98)	3.2 (0.71-9.07)
	Median ABR (IQR)	0.8 (0.24-2.74)	0.4 (0.00-1.29)	0.0 (0.00-0.33)	0.0 (0.00-0.69)	1.2 (0.68-4.00)
	Minimum-maximum	0.00-18.37	0.00-15.41	0.00-12.84	0.00-7.42	0.00-19.16
Total (*N* = 191)	Model-based^b^ ABR (95% CI)	1.4 (1.10-1.74)	0.9 (0.66-1.15)	0.5 (0.35-0.71)	0.4 (0.33-0.60)	2.1 (1.73-2.54)
	Mean ABR (95% CI)	1.5 (0.10-6.39)	0.9 (0.02-5.47)	0.5 (0.00-4.73)	0.5 (0.00-4.74)	2.2 (0.32-7.60)
	Median ABR (IQR)	0.5 (0.00-1.45)	0.2 (0.00-0.86)	0.0 (0.00-0.38)	0.0 (0.00-0.49)	1.0 (0.24-2.62)
	Minimum-maximum	0.00-18.87	0.00-15.41	0.00-12.89	0.00-8.64	0.00-25.90

ABR, annualized bleeding rate; CI, confidence interval; IQR, interquartile range.

a May 12, 2022, for HAVEN 3 and June 29, 2022, for HAVEN 4.

b Negative binomial regression.

Of the 837 treated bleeds experienced in the overall population (*N* = 191), 252 (30.1%) were spontaneous and 585 (69.9%) were traumatic (Supplementary Table S4). A total of 557 (66.5%) bleeds were located in joints, 352 (63.2%) of which were in target joints, 118 (14.1%) bleeds were located in muscle, and 162 (19.4%) bleeds were in other locations.

Nine participants in HAVEN 3 and 3 participants in HAVEN 4 had their emicizumab dose up-titrated to 3 mg/kg QW due to suboptimal bleed control during the study [8]. The calculated model-based ABRs (95% CI) for treated bleeds in the population with a dose up-titration (*n* = 12) was 5.4 (3.58-8.15) before up-titration and 3.5 (2.06-5.99) after up-titration. Of the 3 participants in HAVEN 4 with a dose up-titration, 1 participant subsequently had their dose down-titrated to 3 mg/kg Q2W. The dose was down-titrated after 14 days of up-titration in the absence of further pain and body aches.

In the extended period following the primary analysis of each study, 6 participants in HAVEN 3 changed their dosing regimen, according to their personal preference, to 1 of the other 2 dosing regimens that were approved: 3 participants in arm A, 1 participant in arm B, 1 participant in arm C, and 1 participant in arm D. Of these 6 participants, 4 switched from 1.5 mg/kg QW to 3 mg/kg Q2W. The remaining 2 participants switched from 3 mg/kg Q2W to 6 mg/kg Q4W; however, 1 of these later switched back to 3 mg/kg Q2W. Only 1 participant in HAVEN 4 changed the dosing regimen; it was first changed from 6 mg/kg Q4W to 3 mg/kg Q2W, and then returned to 6 mg/kg Q4W.

### 3.3 Target joint resolution

Over the efficacy period, 60 (48.8%) participants experienced no spontaneous or traumatic bleeds in target joints (Table 3). Of the 306 target joints present at baseline, 291 (95.1%) had no spontaneous or traumatic bleeds in the first 52 weeks after starting emicizumab prophylaxis, meeting the definition for target joint resolution. During the last 52 weeks, 297 (97.1%) target joints had no spontaneous or traumatic bleeds. Additional analyses that included the time after any dose up-titration that occurred were consistent with those excluding this period (Supplementary Table S5).

**Table 3:** Target joint resolution in evaluable participants.

	HAVEN 3 (*N* = 151)	HAVEN 4 (*N* = 40)	Total (*N* = 191)
Evaluable participants^a^ with target joints at baseline, *n* (%)	97 (64.2)	26 (65.0)	123 (64.4)
Target joints at baseline among evaluable participants^a^, *n*	238	68	306
Proportion of evaluable participants^a^ with no spontaneous or traumatic bleeds in target joints, *n* (%)	48 (49.5)	12 (46.2)	60 (48.8)
Target joints with zero spontaneous or traumatic bleeds among target joints from evaluable participants^a^, *n* (%)	168 (70.6)	42 (61.8)	210 (68.6)
Target joints resolved^b^ in the first 52 weeks among target joints from evaluable participants^a^, *n* (%)	226 (95.0)	65 (95.6)	291 (95.1)
Target joints resolved^b^ in the last 52 weeks among target joints from evaluable participants^a^, *n* (%)	230 (96.6)	67 (98.5)	297 (97.1)

a Evaluable participants were those who received ≥12 months of emicizumab (up to dose up-titration, where applicable) until clinical cutoff.

b Target joint resolution was defined as ≤2 spontaneous or traumatic bleeding events in a 52-week period in a joint previously defined as a target joint.

### 3.4 FVIII usage over time

Exploratory analysis of FVIII annualized infusion rate revealed that use of FVIII treatments, including both standard half-life and extended half-life (EHL) products, to treat bleeds generally decreased over time. The mean (95% CI) annualized infusion rate was 3.6 (0.91-9.71) at weeks 1 to 24 (*n* = 186), decreasing to 1.9 (0.21-7.04) at weeks 217 to 240 (*n* = 98), with some fluctuations in between (Supplementary Table S6). The same trend was seen for annualized consumption of FVIII (standard half-life and EHL), with a mean (95% CI) of 110.3 (90.68-132.91) U/kg at weeks 1 to 24 (*n* = 186), decreasing to 53.0 (39.72-69.35) U/kg by weeks 217 to 240 (*n* = 98) (Supplementary Table S6).

### 3.5 Safety

During the studies (median [range] duration of emicizumab exposure: 248.1 [6.1-287.1] weeks), 188 (98.4%) of the 191 participants in the safety population experienced ≥1 AE (Table 4). The most common AEs, seen in ≥10% of participants, were arthralgia (39.3%), injection-site reactions (ISRs; 28.8%), nasopharyngitis (27.7%), headache (23.0%), upper respiratory tract infection (17.8%), back pain (14.7%), influenza (13.1%), and pain in extremity (10.5%).

**Table 4:** Safety summary for study participants from emicizumab initiation until clinical cutoff.

	HAVEN 3 (*N* = 151)	HAVEN 4 (*N* = 40)	Total (*N* = 191)
Total No. of AEs, *n*	1586	445	2031
Participants with ≥1 AE, *n* (%)			
Any AE	148 (98.0)	40 (100.0)	188 (98.4)
Serious AE	35 (23.2)	9 (22.5)	44 (23.0)
AE with fatal outcome	0	0	0
AE leading to withdrawal from treatment	1 (0.7)	0	1 (0.5)
AE leading to dose modification/interruption	2 (1.3)	1 (2.5)	3 (1.6)
Grade ≥3 AE	38 (25.2)	8 (20.0)	46 (24.1)
Treatment-related AE	57 (37.7)	14 (35.0)	71 (37.2)
Injection-site reaction	47 (31.1)	8 (20.0)	55 (28.8)
AE of special interest, *n* (%)			
Systemic hypersensitivity/anaphylactic/anaphylactoid reaction	0	0	0
Thromboembolic event	2^a^ (1.3)	0	2 (1.0)
Thrombotic microangiopathy	0	0	0

AE, adverse event.

a In 1 participant, a subsequent medical review revealed no evidence of thrombosis.

A total of 185 treatment-related AEs were reported by 71 (37.2%) participants across the 2 studies. The most commonly reported treatment-related AE was ISR, which 55 (28.8%) participants experienced. Other treatment-related AEs experienced by ≥2 participants were headaches (*n* = 5 [2.6%]), rash (*n* = 3 [1.6%]), pruritus (*n* = 2 [1.0%]), and nausea (*n* = 2 [1.0%]).

A total of 142 ISRs were reported, all of which were classed as related to the study treatment. Of these, 140 (98.6%) were grade 1 in intensity and 2 (1.4%) were grade 2. None was classed as an SAE. Overall, 120 (84.5%) of the ISRs resolved within 4 days. Of the 55 participants who experienced an ISR, 6 (10.9%) received additional treatment (including oral/local antihistamine or corticosteroid cream). All ISRs resolved without alteration of emicizumab treatment. The proportion of participants who experienced an ISR decreased over time (Supplementary Figure S3), with only an occasional event occurring after participants had received 52 doses.

Overall, 44 (23.0%) participants reported an SAE, none of which were deemed emicizumab-related. No thrombotic microangiopathies were reported in either study. Two potential TEs were reported in HAVEN 3: a myocardial infarction (grade 3) in one participant and an acute coronary syndrome (grade 2) in another. The investigator in each case considered these events to be unrelated to emicizumab. The participant with the myocardial infarction (arm B), aged 65 years, had a history of hypertension and received episodic FVIII treatment prior to study entry. He was admitted to hospital for a scheduled spinal laminectomy, which was performed on study day 1015. Two days later, he was noted to be short of breath with elevated troponin. He was diagnosed with a non-ST segment elevation myocardial infarction on study day 1018. Upon undergoing coronary angiography, he was found to have severe triple-vessel coronary artery disease, which was previously undiagnosed. He then underwent bypass graft surgery, mitral valve repair, and aortic valve replacement on study day 1028. On study day 1051, the myocardial infarction was considered resolved. The participant with the acute coronary syndrome (arm D), aged 65 years, had a history of hypertension and received FVIII prophylactic treatment prior to study entry. He was hospitalized after reporting chest pains and was diagnosed with moderate acute coronary syndrome on study day 239. He was subsequently treated with medication and underwent a percutaneous coronary intervention. The acute coronary syndrome was considered resolved on study day 611. Although the search criteria identified this case as a potential TE, further medical review of the clinical details for this case revealed no evidence of thrombosis. Emicizumab treatment was maintained throughout for both participants.

Overall, 1 (0.5%) participant withdrew from treatment due to multiple low-grade AEs, which was previously reported in the primary analysis [6]. This participant reported AEs of headache, lethargy, depressed mood, insomnia, nightmares, alopecia, and pruritus, all of which were grade 1 to 2 in severity. Three (1.6%) participants reported an AE that led to dose modification or interruption (gastroenteritis, synovitis, and a head injury, respectively). The participant with gastroenteritis (HAVEN 3, arm A) experienced symptoms that prevented him from attending the clinic to receive his emicizumab dose. He was able to resume treatment 7 days later. The participant with synovitis (HAVEN 3, arm D) had his dose increased due to the AE, which was classified as serious, and he also fulfilled the criteria for up-titration. The SAE resolved after 26 days with treatment. The head injury was experienced by a participant in HAVEN 4 who suffered a fall and injured his head. He was hospitalized, and the injury was resolved on the day of admission. This participant was classified as having an AE that led to dose interruption/modification since the study treatment could not be given to him on the scheduled date as he was being evaluated and treated in hospital. The study treatment was given at home 2 days later than scheduled.

### 3.6 Immunogenicity

There was no development of *de novo* FVIII inhibitors reported in either HAVEN 3 or 4. At the time of the LPLV, 8 (4.2%) participants had tested positive for ADAs across the 2 studies; however, all these participants tested negative for ADAs by their last assessment. Of these 8 participants, 5 tested positive for *in vitro* neutralizing ADAs; 3 participants presented with an ADA on a single occasion, while the other 2 presented with long-lasting ADAs. The presence of the ADAs showed no evidence of impact on the efficacy or safety profile of emicizumab [10], with a model-based ABR (95% CI) of 0.6 (0.28-1.14) for treated bleeds in the 8 participants who tested positive for ADAs. A decrease in emicizumab concentration was not seen for any of the participants who tested positive for ADAs. None of the 8 participants discontinued emicizumab prophylaxis during their respective studies.

## 4 Discussion

The final analyses of the HAVEN 3 and 4 studies provide insight into the long-term efficacy and safety of emicizumab prophylaxis in people with HA without FVIII inhibitors. The overall ABRs in both studies remained consistently low throughout long-term follow-up, with no unexpected or new safety signals observed.

The BMQ compliance rate remained high (92.9%), despite the long-term follow-up of nearly 5 years. ABRs were low across the studies, with most improvement seen in the first 6 months and stabilizing thereafter. This confirmed consistent efficacy of emicizumab over time and is corroborated by the proportion of participants with zero treated bleeds remaining high throughout the 2 studies. Approximately two-thirds of the bleeds experienced during the studies were traumatic, with joints being the predominant location. The participants on prior FVIII prophylaxis (HAVEN 3, arm D) also showed decreased ABRs when switched to emicizumab prophylaxis [6].

The low ABRs for target joint bleeds translated into more than 95% of the target joints present at baseline resolving during the first year of treatment with emicizumab. This indicates that emicizumab prophylaxis, given over longer periods of time, may be able to maintain or improve joint health, potentially having a notable impact on quality of life [[12], [13], [14], [15]].

The median (IQR) ABRs for all bleeds demonstrated during long-term emicizumab prophylaxis, 1.0 (0.24-2.62), were similar to those that have been reported for people with HA receiving EHL FVIII prophylaxis, ranging from 0 to 1.49 [[16], [17], [18]]; however, these were confirmed over a shorter follow-up time (<5 years) than that in the present analysis. The proportions of participants with zero bleeds varied in different trials and were not reported for the same time periods; hence, they are hard to compare directly [17,19,20]. A recent systematic literature review on people with HA receiving FVIII prophylaxis, however, reported a median (IQR) of 39.6% (27.1%-48.0%) of participants with zero bleeds overall and a value of 36.0% (24.5%-46.4%) for those receiving EHL FVIII [21]. This compares with 47.3% of participants experiencing zero bleeds during weeks 1 to 24 and 75.8% during weeks 217 to 240 in HAVEN 3 and 4. This high rate of zero bleeds during long-term follow-up is a positive outcome, given that the latest edition of the World Federation of Hemophilia guidelines highlights the importance of optimizing prophylactic therapy to achieve the ultimate aim of “zero bleeds” [1].

Gene therapy has also shown the potential for similarly low ABRs to those reported in the present analysis; however, a degree of variability in outcomes has been observed, and long-term data indicating duration of response are currently limited [[22], [23], [24], [25]].

No new safety signals were observed during this long-term follow-up of people with HA treated with emicizumab prophylaxis. After a median observation period of 262.3 weeks for HAVEN 3 and 251.9 weeks for HAVEN 4, the long-term safety profile of emicizumab prophylaxis was favorable and consistent with the findings of the primary analyses, the other HAVEN studies, and the STASEY safety study in people with HA with inhibitors [6,7,[26], [27], [28], [29], [30]]. There were no reported fatalities in the participants without FVIII inhibitors enrolled in HAVEN 3 or 4. Two individuals in the HAVEN 3 study experienced a TE: a myocardial infarction and an acute coronary syndrome, both deemed unrelated to emicizumab.

A total of 8 (4.2%) participants in HAVEN 3 and 4 tested positive for ADAs during their respective studies, with no evidence of clinically significant neutralizing ADAs. This is in line with the small proportions of ADA-positive participants in the other HAVEN studies, STASEY, and HOHOEMI, which ranged from 0% to 12.5% [10]. Only a few other cases have been reported in the literature, which could indicate that the overall development of ADAs remains low in people with HA receiving emicizumab [31,32]. The presence of ADAs showed no evidence of impact on safety and efficacy in this analysis, and all ADAs had spontaneously resolved by the time of the final analysis.

At study completion, a high proportion of participants continued using emicizumab, either via a posttrial access program or by switching to commercial emicizumab. This is in agreement with the high proportion of participants in HAVEN 3 (94%) and HAVEN 4 (100%), who reported that they preferred emicizumab over their prior treatments when completing the EmiPref questionnaire at week 17 of their respective study [33].

Outside of the clinical trial setting, the ongoing collection of real-world data provides further information on the benefit-risk profile of long-term emicizumab, particularly in the context of newer therapies, including other nonreplacement therapies, EHL FVIII products, and gene therapy.

### 4.1 Limitations

There were some limitations to the present analysis. First, data on bleeds relied on the use of a self-reporting tool by participants; hence, assessments of bleeds and whether treatment was required were subjective. Moreover, as bleeds were self-reported, reporting fatigue (participants being less vigilant in reporting) may have occurred; however, maintenance of high compliance over time indicated that this would have had minimal impact on these studies. Attrition, resulting from participants who switched to commercial emicizumab, associated with long-term analyses, is a further potential limitation.

## 5 Conclusion

With nearly 5 years of follow-up and 729.3 participant-years of exposure, these data build on previous findings of a favorable long-term benefit-risk profile for emicizumab prophylaxis in people with HA without inhibitors, with consistent bleed prevention and no new or unexpected safety signals.
