# Supplementary material for: Long-term outcomes with emicizumab in hemophilia A without inhibitors: results from the HAVEN 3 and 4 studies
Source: Res Pract Thromb Haemost. 2024 Mar 1;8(2):102364. doi: 10.1016/j.rpth.2024.102364 (PMC10978536; doi:10.1016/j.rpth.2024.102364)

**Long-term outcomes with emicizumab in hemophilia A without inhibitors: results from the HAVEN 3 and 4 studies**

**Authors:** Johnny Mahlangu, Víctor Jiménez-Yuste, Giuliana Ventriglia, Markus Niggli, Simona Barlera, Cédric Hermans, Michaela Lehle, Pratima Chowdary, Lyle Jew, Jerzy Windyga, Laurent Frenzel, Christophe Schmitt, Giancarlo Castaman, Steven W. Pipe

**TABLE S1** Bleeding events in participants in HAVEN 3 (Arm D) who had previously received FVIII prophylaxis in a non-interventional study (*N* = 48).

|  | **Efficacy observation period**  **Mean (SD), weeks** | **Treated bleeds**  **Model-based ABR (95% CI)** | **All bleeds**  **Model-based ABR (95% CI)** |
| --- | --- | --- | --- |
| **FVIII prophylaxis in NIS*** | 31.1 (7.88) | 4.8 (3.22-7.09) | 8.9 (5.72-13.87) |
| **Emicizumab prophylaxis in HAVEN 3^†^ (Arm D^‡^)** | 33.2 (7.14) | 1.5 (0.98-2.33) | 3.3 (2.17-5.06) |

*Kruse-Jarres et al. *Haemophilia* 2019; this only included data on the 48 participants that transferred from the NIS to Arm D of HAVEN 3; ^†^Until clinical cut-off, Sep 15, 2017; ^‡^Participants received emicizumab 1.5 mg/kg QW.

ABR, annualized bleed rate; CI, confidence interval; F, factor; NIS, non-interventional study; QW, once weekly; SD, standard deviation.

**TABLE S2** Calculated ABRs and proportion of participants with 0 and 0-3 bleeds over consecutive 24-week intervals among total participants.

|  | **Treated bleeds** | **All bleeds** | **Treated spontaneous bleeds** | **Treated joint bleeds** | **Treated target joint bleeds** |
| --- | --- | --- | --- | --- | --- |
| **Weeks 1-24, *N* = 188**  Mean ABR [95% CI]  Participants with 0 bleeds, *n* (%) [95% CI]  Participants with 0-3 bleeds, *n* (%) [95% CI] | 2.0 [0.23-7.15]  117 (62.2) [54.89-69.19]  174 (92.6) [87.82-95.87] | 3.4 [0.82-9.43]  89 (47.3) [40.03-54.74]  160 (85.1) [79.20-89.87] | 0.6 [0.00-4.95]  155 (82.4) [76.24-87.60]  184 (97.9) [94.64-99.42] | 1.4 [0.08-6.19]  136 (72.3) [65.36-78.60]  178 (94.7) [90.44-97.42] | 0.9 [0.01-5.34]  156 (83.0) [76.83-88.06]  183 (97.3) [93.90-99.13] |
| **Weeks 25-48, *N* = 182**  Mean ABR [95% CI]  Participants with 0 bleeds, *n* (%) [95% CI]  Participants with 0-3 bleeds, *n* (%) [95% CI] | 1.1 [0.04-5.76] 133 (73.1) [66.01-79.37]  176 (96.7) [92.96-98.78] | 1.9 [0.22-7.08]  105 (57.7) [50.16-64.97]  168 (92.3) [87.43-95.73] | 0.3 [0.00-4.31]  162 (89.0) [83.54-93.16]  181 (99.5) [96.98-99.99] | 0.6 [0.00-4.90]  154 (84.6) [78.54-89.53]  180 (98.9) [96.09-99.87] | 0.4 [0.00-4.52]  164 (90.1) [84.82-94.03]  180 (98.9) [96.09-99.87] |
| **Weeks 49-72, *N* = 176**  Mean ABR [95% CI]  Participants with 0 bleeds, *n* (%) [95% CI]  Participants with 0-3 bleeds, *n* (%) [95% CI] | 1.3 [0.06-6.02]  130 (73.9) [66.72-80.19]  168 (95.5) [91.24-98.02] | 1.9 [0.20-7.01]  108 (61.4) [53.74-68.59]  164 (93.2) [88.39-96.43] | 0.4 [0.00-4.43]  153 (86.9) [81.04-91.53]  176 (100.0) [97.93-100.00] | 0.8 [0.01-5.20]  148 (84.1) [77.83-89.16]  173 (98.3) [95.10-99.65] | 0.5 [0.00-4.73]  160 (90.9) [85.66-94.71]  173 (98.3) [95.10-99.65] |
| **Weeks 73-96, *N* = 158**  Mean ABR [95% CI]  Participants with 0 bleeds, *n* (%) [95% CI]  Participants with 0-3 bleeds, *n* (%) [95% CI] | 0.9 [0.02-5.46]  127 (80.4) [73.32-86.26]  155 (98.1) [94.55-99.61] | 1.4 [0.08-6.21]  111 (70.3) [62.47-77.25]  153 (96.8) [92.77-98.96] | 0.2 [0.00-4.14]  150 (94.9) [90.27-97.79]  157 (99.4) [96.52-99.98] | 0.6 [0.00-4.79]  138 (87.3) [81.13-92.09]  155 (98.1) [94.55-99.61] | 0.4 [0.00-4.43]  145 (91.8) [86.34-95.55]  155 (98.1) [94.55-99.61] |
| **Weeks 97-120, *N* = 128**  Mean ABR [95% CI]  Participants with 0 bleeds, *n* (%) [95% CI]  Participants with 0-3 bleeds, *n* (%) [95% CI] | 1.0 [0.02-5.52]  100 (78.1) [69.96-84.95]  124 (96.9) [92.19-99.14] | 1.3 [0.07-6.10]  90 (70.3) [61.60-78.06]  123 (96.1) [91.12-98.72] | 0.3 [0.00-4.21]  116 (90.6) [84.20-95.06]  128 (100.0) [97.16-100.00] | 0.7 [0.00-5.07]  106 (82.8) [75.14-88.90]  125 (97.7) [93.30-99.51] | 0.4 [0.00-4.44]  115 (89.8) [83.26-94.48]  127 (99.2) [95.72-99.98] |
| **Weeks 121-144, *N* = 118**  Mean ABR [95% CI]  Participants with 0 bleeds, *n* (%) [95% CI]  Participants with 0-3 bleeds, *n* (%) [95% CI] | 0.9 [0.02-5.47]  92 (78.0) [69.41-85.07]  114 (96.6) [91.55-99.07] | 1.1 [0.04-5.82]  84 (71.2) [62.13-79.15]  114 (96.6) [91.55-99.07] | 0.4 [0.00-4.46]  105 (89.0) [81.90-94.00]  117 (99.2) [95.37-99.98] | 0.6 [0.00-4.91]  102 (86.4) [78.92-92.05]  115 (97.5) [92.75-99.47] | 0.4 [0.00-4.53]  105 (89.0) [81.90-94.00]  117 (99.2) [95.37-99.98] |
| **Weeks 145-168, *N* = 113**  Mean ABR [95% CI]  Participants with 0 bleeds, *n* (%) [95% CI]  Participants with 0-3 bleeds, *n* (%) [95% CI] | 1.3 [0.08-6.16]  83 (73.5) [64.32-81.32]  109 (96.5) [91.18-99.03] | 1.6 [0.13-6.54]  76 (67.3) [57.79-75.79]  107 (94.7) [88.80-98.03] | 0.6 [0.00-4.92]  94 (83.2) [74.99-89.56]  112 (99.1) [95.17-99.98] | 1.0 [0.03-5.61]  89 (78.8) [70.07-85.89]  110 (97.3) [92.44-99.45] | 0.6 [0.00-4.85]  98 (86.7) [79.05-92.38]  111 (98.2) [93.75-99.78] |
| **Weeks 169-192, *N* = 108**  Mean ABR [95% CI]  Participants with 0 bleeds, *n* (%) [95% CI]  Participants with 0-3 bleeds, *n* (%) [95% CI] | 1.3 [0.07-6.06]  92 (85.2) [77.06-91.29]  103 (95.4) [89.53-98.48] | 1.7 [0.15-6.73]  84 (77.8) [68.76-85.21]  102 (94.4) [88.30-97.93] | 0.3 [0.00-4.22]  101 (93.5) [87.10-97.35]  107 (99.1) [94.95-99.98] | 1.0 [0.03-5.58]  97 (89.8) [82.51-94.80]  104 (96.3) [90.79-98.98] | 0.7 [0.00-5.01]  103 (95.4) [89.53-98.48]  106 (98.1) [93.47-99.77] |
| **Weeks 193-216, *N* = 106**  Mean ABR [95% CI]  Participants with 0 bleeds, *n* (%) [95% CI]  Participants with 0-3 bleeds, *n* (%) [95% CI] | 1.2 [0.06-5.97]  82 (77.4) [68.21-84.92]  102 (96.2) [90.62-98.96] | 1.5 [0.11-6.41]  77 (72.6) [63.13-80.85]  101 (95.3) [89.33-98.45] | 0.2 [0.00-4.15]  98 (92.5) [85.67-96.69]  106 (100.0) [96.58-100.00] | 0.8 [0.01-5.22]  90 (84.9) [76.65-91.12]  104 (98.1) [93.35-99.77] | 0.6 [0.00-4.89]  95 (89.6) [82.19-94.70]  105 (99.1) [94.86-99.98] |
| **Weeks 217-240, *N* = 99**  Mean ABR [95% CI]  Participants with 0 bleeds, *n* (%) [95% CI]  Participants with 0-3 bleeds, *n* (%) [95% CI] | 0.8 [0.01-5.28]  78 (78.8) [69.42-86.36]  96 (97.0) [91.40-99.37] | 1.0 [0.03-5.63]  75 (75.8) [66.11-83.81]  95 (96.0) [89.98-98.89] | 0.2 [0.00-4.05]  92 (92.9) [85.97-97.11]  99 (100.0) [96.34-100.00] | 0.5 [0.00-4.60]  85 (85.9) [77.41-92.05]  98 (99.0) [94.50-99.97] | 0.2 [0.00-4.09]  94 (94.9) [88.61-98.34]  98 (99.0) [94.50-99.97] |
| **Weeks 241-264, *N* = 67**  Mean ABR [95% CI]  Participants with 0 bleeds, *n* (%) [95% CI]  Participants with 0-3 bleeds, *n* (%) [95% CI] | 0.9 [0.01-5.36]  53 (79.1) [67.43-88.08]  65 (97.0) [89.63-99.64] | 1.0 [0.02-5.53]  50 (74.6) [62.51-84.47]  65 (97.0) [89.63-99.64] | 0.2 [0.00-4.15]  61 (91.0) [81.52-96.64]  67 (100.0) [94.64-100.00] | 0.6 [0.00-4.89]  57 (85.1) [74.26-92.60]  66 (98.5) [91.96-99.96] | 0.4 [0.00-4.53]  59 (88.1) [77.82-94.70]  66 (98.5) [91.96-99.96] |

ABR, annualized bleed rate; CI, confidence interval.

**TABLE S3** Calculated mean ABRs and proportion of participants with 0-3 bleeds over consecutive 52-week intervals among total participants.

|  | **Treated bleeds** | **All bleeds** | **Treated spontaneous bleeds** | **Treated joint bleeds** | **Treated target joint bleeds** |
| --- | --- | --- | --- | --- | --- |
| **Weeks 1-52, *N* = 182**  ABR, mean [95% CI]  Participants with 0 bleeds, *n* (%) [95% CI]  Participants with 0-3 bleeds, *n* (%) [95% CI] | 1.4 [0.08-6.22]  92 (50.5) [43.05-58.03]  165 (90.7) [85.47-94.46] | 2.5 [0.40-7.96]  64 (35.2) [28.25-42.57]  143 (78.6) [71.89-84.30] | 0.4 [0.00-4.47]  142 (78.0) [71.30-83.81]  178 (97.8) [94.47-99.40] | 0.9 [0.02-5.39]  120 (65.9) [58.56-72.78]  171 (94.0) [89.44-96.94] | 0.6 [0.00-4.84]  144 (79.1) [72.49-84.78]  177 (97.3) [93.71-99.10] |
| **Weeks 53-104, *N* = 144**  ABR, mean [95% CI]  Participants with 0 bleeds, *n* (%) [95% CI]  Participants with 0-3 bleeds, *n* (%) [95% CI] | 1.0 [0.02-5.52]  88 (61.1) [52.64-69.12]  134 (93.1) [87.60-96.62] | 1.4 [0.09-6.31]  71 (49.3) [40.88-57.76]  132 (91.7) [85.90-95.62] | 0.3 [0.00-4.20]  121 (84.0) [77.00-89.60]  143 (99.3) [96.19-99.98] | 0.6 [0.00-4.91]  109 (75.7) [67.85-82.45]  139 (96.5) [92.08-98.86] | 0.4 [0.00-4.51]  122 (84.7) [77.79-90.17]  140 (97.2) [93.04-99.24] |
| **Weeks 105-156, *N* = 117** ABR, mean [95% CI]  Participants with 0 bleeds, *n* (%) [95% CI]  Participants with 0-3 bleeds, *n* (%) [95% CI] | 1.0 [0.03-5.59]  76 (65.0) [55.59-73.55]  107 (91.5) [84.84-95.83] | 1.3 [0.06-6.02]  63 (53.8) [44.39-63.10]  105 (89.7) [82.77-94.59] | 0.5 [0.00-4.61]  92 (78.6) [70.09-85.67]  113 (96.6) [91.48-99.06] | 0.7 [0.01-5.12]  84 (71.8) [62.73-79.72]  110 (94.0) [88.06-97.56] | 0.4 [0.00-4.54]  95 (81.2) [72.93-87.82]  114 (97.4) [92.69-99.47] |
| **Weeks 157-208, *N* = 106**  ABR, mean [95% CI]  Participants with 0 bleeds, *n* (%) [95% CI]  Participants with 0-3 bleeds, *n* (%) [95% CI] | 1.4 [0.09-6.24]  74 (69.8) [60.13-78.35]  95 (89.6) [82.19-94.70] | 1.7 [0.16-6.77]  64 (60.4) [50.41-69.75]  92 (86.8) [78.83-92.59] | 0.3 [0.00-4.34]  89 (84.0) [75.57-90.37]  103 (97.2) [91.95-99.41] | 1.0 [0.03-5.63]  82 (77.4) [68.21-84.92]  101 (95.3) [89.33-98.45] | 0.8 [0.01-5.18]  90 (84.9) [76.65-91.12]  103 (97.2) [91.95-99.41] |
| **Weeks 209-260, *N* = 78**  ABR, mean [95% CI]  Participants with 0 bleeds, *n* (%) [95% CI]  Participants with 0-3 bleeds, *n* (%) [95% CI] | 0.8 [0.01-5.19]  55 (70.5) [59.11-80.30]  74 (94.9) [87.39-98.59] | 0.9 [0.02-5.38]  48 (61.5) [49.83-72.34]  73 (93.6) [85.67-97.89] | 0.2 [0.00-4.08]  66 (84.6) [74.67-91.79]  78 (100.0) [95.38-100.00] | 0.5 [0.00-4.68]  61 (78.2) [67.41-86.76]  75 (96.2) [89.17-99.20] | 0.3 [0.00-4.29]  68 (87.2) [77.68-93.68]  76 (97.4) [91.04-99.69] |

ABR, annualized bleed rate; CI, confidence interval.

**TABLE S4** Types and locations of treated bleeds from emicizumab initiation until clinical cut-off.

|  | **HAVEN 3 (*N* = 151)** | **HAVEN 4 (*N* = 40)** | **Total (*N* = 191)** |
| --- | --- | --- | --- |
| **Total number of bleeds, *n*** | 575 | 262 | 837 |
| **Number of bleeds per bleed type, *n* (%)** |  |  |  |
| Spontaneous | 187 (32.5) | 65 (24.8) | 252 (30.1) |
| Traumatic* | 388 (67.5) | 197 (75.2) | 585 (69.9) |
| **Number of bleeds per bleed location, *n* (%)** |  |  |  |
| Joint | 375 (65.2) | 182 (69.5) | 557 (66.5) |
| Muscle | 78 (13.6) | 40 (15.3) | 118 (14.1) |
| Other | 122 (21.2) | 40 (15.3) | 162 (19.4) |

*A bleed for which there was an identifiable cause.

**TABLE S5** Target joint resolution in evaluable participants, including the period after dose up-titration.

|  | **HAVEN 3 (*N* = 151)** | **HAVEN 4 (*N* = 40)** | **Total (*N* = 191)** |
| --- | --- | --- | --- |
| Evaluable participants* with target joints at baseline, *n* (%) | 100 (66.2) | 28 (70.0) | 128 (67.0) |
| Target joints at baseline among evaluable participants*, *n* | 250 | 73 | 323 |
| Proportion of evaluable participants* with no spontaneous or traumatic bleeds in target joints, *n* (%) | 49 (49.0) | 12 (42.9) | 61 (47.7) |
| Target joints with zero spontaneous or traumatic bleeds among target joints from evaluable participants*, *n* (%) | 176 (70.4) | 43 (58.9) | 219 (67.8) |
| Target joints resolved^†^ in the first 52 weeks among target joints from evaluable participants*, *n* (%) | 240 (96.0) | 70 (95.9) | 310 (96.0) |
| Target joints resolved^†^ in the last 52 weeks among target joints from evaluable participants*, *n* (%) | 246 (98.4) | 72 (98.6) | 318 (98.5) |

*Evaluable participants were participants who received ≥12 months of emicizumab until clinical cut-off. ^†^Target joint resolution was defined as
≤2 spontaneous or traumatic bleeding events in a 52-week period in a joint previously defined as a target joint.

**TABLE S6** Annualized FVIII infusion rate and FVIII consumption over 24-week intervals.

|  | **FVIII annualized infusion rate** | | | **FVIII consumption (U/kg)** | | |
| --- | --- | --- | --- | --- | --- | --- |
|  | **Treatment for bleed** | **Preventative dose prior to activity** | **Total** | **Treatment for bleed** | **Preventative  dose prior to  activity** | **Total** |
| **Weeks 1-24, *N* = 186** |  |  |  |  |  |  |
| Mean (95% CI) | 3.3 (0.76-9.23) | 0.3 (0.00-4.35) | 3.6 (0.91-9.71) | 101.6 (82.83-123.41) | 8.7 (3.90-16.66) | 110.3 (90.68-132.91) |
| Median (IQR) | 0.0 (0.00-2.17) | 0.0 (0.00-0.00) | 0.0 (0.00-4.35) | 0.0 (0.00-77.37) | 0.0 (0.00-0.00) | 0.0 (0.00-103.53) |
| **Weeks 25-48, *N* = 182** |  |  |  |  |  |  |
| Mean (95% CI) | 3.7 (0.92-9.76) | 0.1 (0.00-3.89) | 3.8 (0.97-9.90) | 107.6 (88.28-130.01) | 3.3 (0.77-9.27) | 111.0 (91.30-133.66) |
| Median (IQR) | 0.0 (0.00-2.17) | 0.0 (0.00-0.00) | 0.0 (0.00-2.17) | 0.0 (0.00-49.26) | 0.0 (0.00-0.00) | 0.0 (0.00-49.26) |
| **Weeks 49-72, *N* = 175** |  |  |  |  |  |  |
| Mean (95% CI) | 3.4 (0.81-9.41) | 0.1 (0.00-3.84) | 3.5 (0.85-9.52) | 100.4 (81.74-122.09) | 2.9 (0.59-8.66) | 103.3 (84.38-125.30) |
| Median (IQR) | 0.0 (0.00-2.17) | 0.0 (0.00-0.00) | 0.0 (0.00-2.17) | 0.0 (0.00-51.76) | 0.0 (0.00-0.00) | 0.0 (0.00-62.47) |
| **Weeks 73-96, *N* = 154** |  |  |  |  |  |  |
| Mean (95% CI) | 2.1 (0.28-7.43) | 0.0 (0.00-3.75) | 2.2 (0.29-7.48) | 62.9 (48.30-80.46) | 0.3 (0.00-4.29) | 63.2 (48.56-80.79) |
| Median (IQR) | 0.0 (0.00-0.00) | 0.0 (0.00-0.00) | 0.0 (0.00-0.00) | 0.0 (0.00-0.00) | 0.0 (0.00-0.00) | 0.0 (0.00-0.00) |
| **Weeks 97-120, *N* = 127** |  |  |  |  |  |  |
| Mean (95% CI) | 2.0 (0.24-7.23) | 0.1 (0.00-3.87) | 2.1 (0.27-7.36) | 56.7 (42.92-73.52) | 1.9 (0.22-7.10) | 58.6 (44.59-75.69) |
| Median (IQR) | 0.0 (0.00-0.00) | 0.0 (0.00-0.00) | 0.0 (0.00-0.00) | 0.0 (0.00-0.00) | 0.0 (0.00-0.00) | 0.0 (0.00-0.00) |
| **Weeks 121-144, *N* = 117** |  |  |  |  |  |  |
| Mean (95% CI) | 2.6 (0.46-8.19) | 0.2 (0.00-4.03) | 2.8 (0.53-8.45) | 73.7 (57.83-92.55) | 3.8 (0.99-9.94) | 77.5 (61.19-96.76) |
| Median (IQR) | 0.0 (0.00-0.00) | 0.0 (0.00-0.00) | 0.0 (0.00-0.00) | 0.0 (0.00-0.00) | 0.0 (0.00-0.00) | 0.0 (0.00-0.00) |
| **Weeks 145-168, *N* = 113** |  |  |  |  |  |  |
| Mean (95% CI) | 2.7 (0.51-8.36) | 0.2 (0.00-4.01) | 2.9 (0.57-8.60) | 79.3 (62.77-98.74) | 2.7 (0.51-8.36) | 82.0 (65.20-101.76) |
| Median (IQR) | 0.0 (0.00-2.17) | 0.0 (0.00-0.00) | 0.0 (0.00-2.17) | 0.0 (0.00-42.35) | 0.0 (0.00-0.00) | 0.0 (0.00-42.35) |
| **Weeks 169-192, *N* = 107** |  |  |  |  |  |  |
| Mean (95% CI) | 2.1 (0.26-7.31) | 0.6 (0.00-4.88) | 2.7 (0.48-8.25) | 62.1 (47.61-79.57) | 13.3 (7.12-22.57) | 75.4 (59.30-94.40) |
| Median (IQR) | 0.0 (0.00-0.00) | 0.0 (0.00-0.00) | 0.0 (0.00-0.00) | 0.0 (0.00-0.00) | 0.0 (0.00-0.00) | 0.0 (0.00-0.00) |
| **Weeks 193-216, *N* = 104** |  |  |  |  |  |  |
| Mean (95% CI) | 1.9 (0.22-7.10) | 0.1 (0.00-3.99) | 2.1 (0.26-7.33) | 54.2 (40.75-70.70) | 3.8 (1.01-10.01) | 58.1 (44.09-75.04) |
| Median (IQR) | 0.0 (0.00-0.00) | 0.0 (0.00-0.00) | 0.0 (0.00-0.00) | 0.0 (0.00-0.00) | 0.0 (0.00-0.00) | 0.0 (0.00-0.00) |
| **Weeks 217-240, *N* = 98** |  |  |  |  |  |  |
| Mean (95% CI) | 1.6 (0.14-6.61) | 0.3 (0.00-4.23) | 1.9 (0.21-7.04) | 45.3 (33.05-60.52) | 7.8 (3.29-15.43) | 53.0 (39.72-69.35) |
| Median (IQR) | 0.0 (0.00-0.00) | 0.0 (0.00-0.00) | 0.0 (0.00-2.17) | 0.0 (0.00-0.00) | 0.0 (0.00-0.00) | 0.0 (0.00-39.53) |
| **Weeks 241-264, *N* = 61** |  |  |  |  |  |  |
| Mean (95% CI) | 1.1 (0.03-5.69) | 0.0 (NA-3.69) | 1.1 (0.03-5.69) | 33.4 (23.05-46.82) | 0.0 (NA-3.69) | 33.4 (23.05-46.82) |
| Median (IQR) | 0.0 (0.00-0.00) | 0.0 (0.00-0.00) | 0.0 (0.00-0.00) | 0.0 (0.00-0.00) | 0.0 (0.00-0.00) | 0.0 (0.00-0.00) |

Data exclude preventative dose prior to procedure/surgery; FVIII includes standard and extended half-life products; for those participants who were on previous prophylactic FVIII regimens prior to study entry, FVIII given in the first week of treatment is excluded.

CI, confidence interval; F, factor; IQR, interquartile range; NA, not applicable.

**FIGURE S1** Mean ABRs for treated bleeds over 52-week intervals.


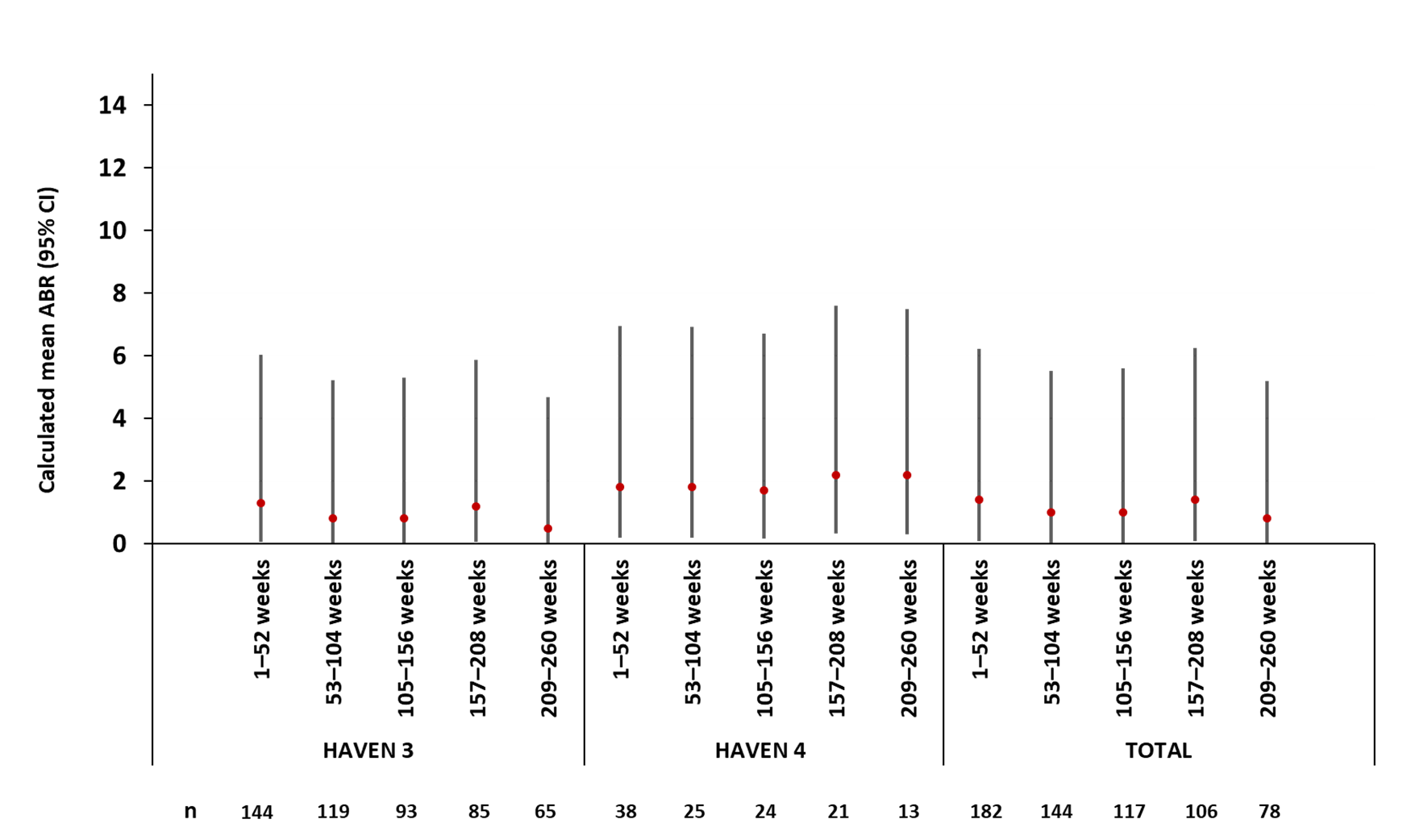


ABR, annualized bleed rate; CI, confidence interval.

**FIGURE S2** Proportion of participants with zero treated bleeds over 52-week intervals.


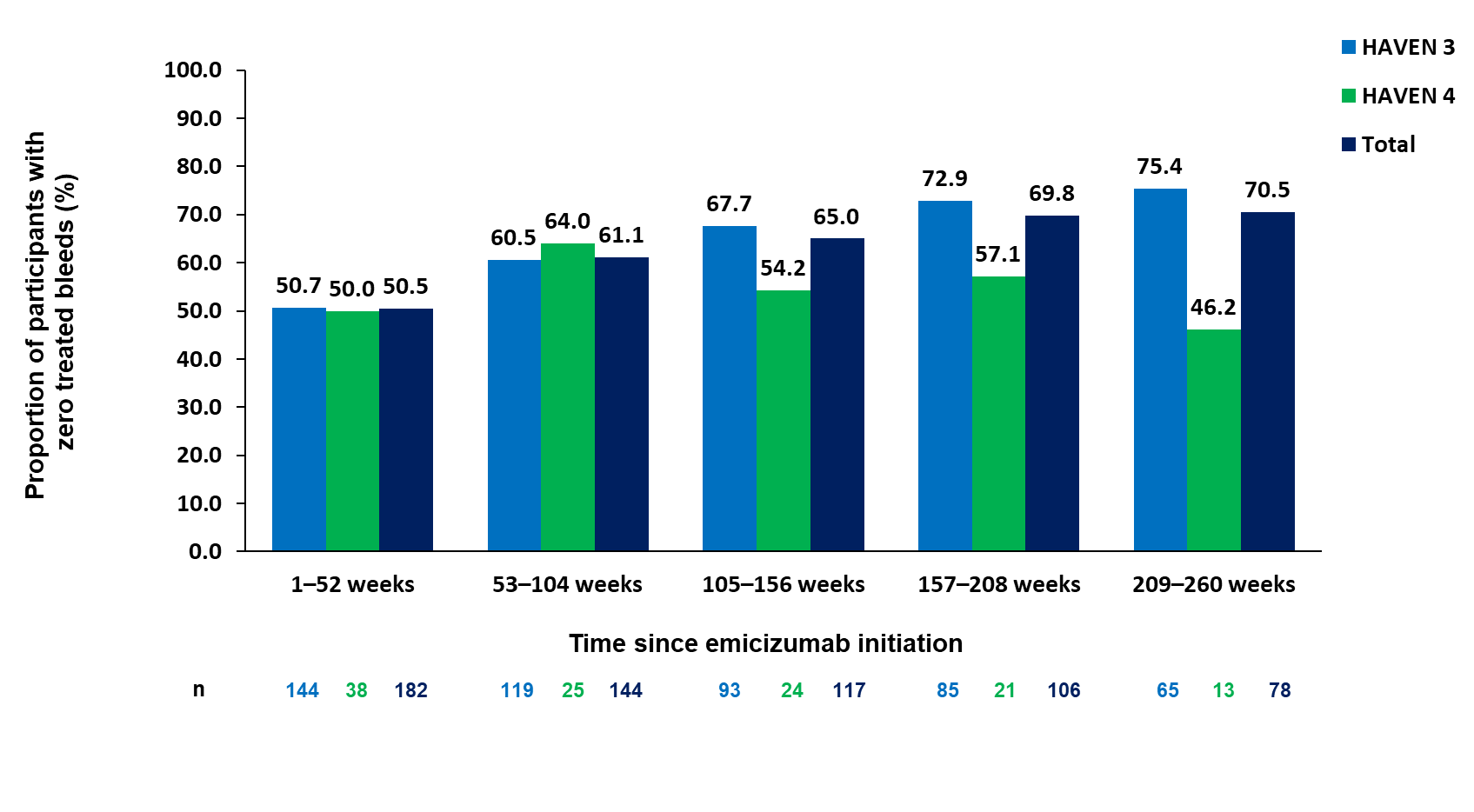


**FIGURE S3** Proportion of participants in total population who experienced an ISR according to number of emicizumab doses.


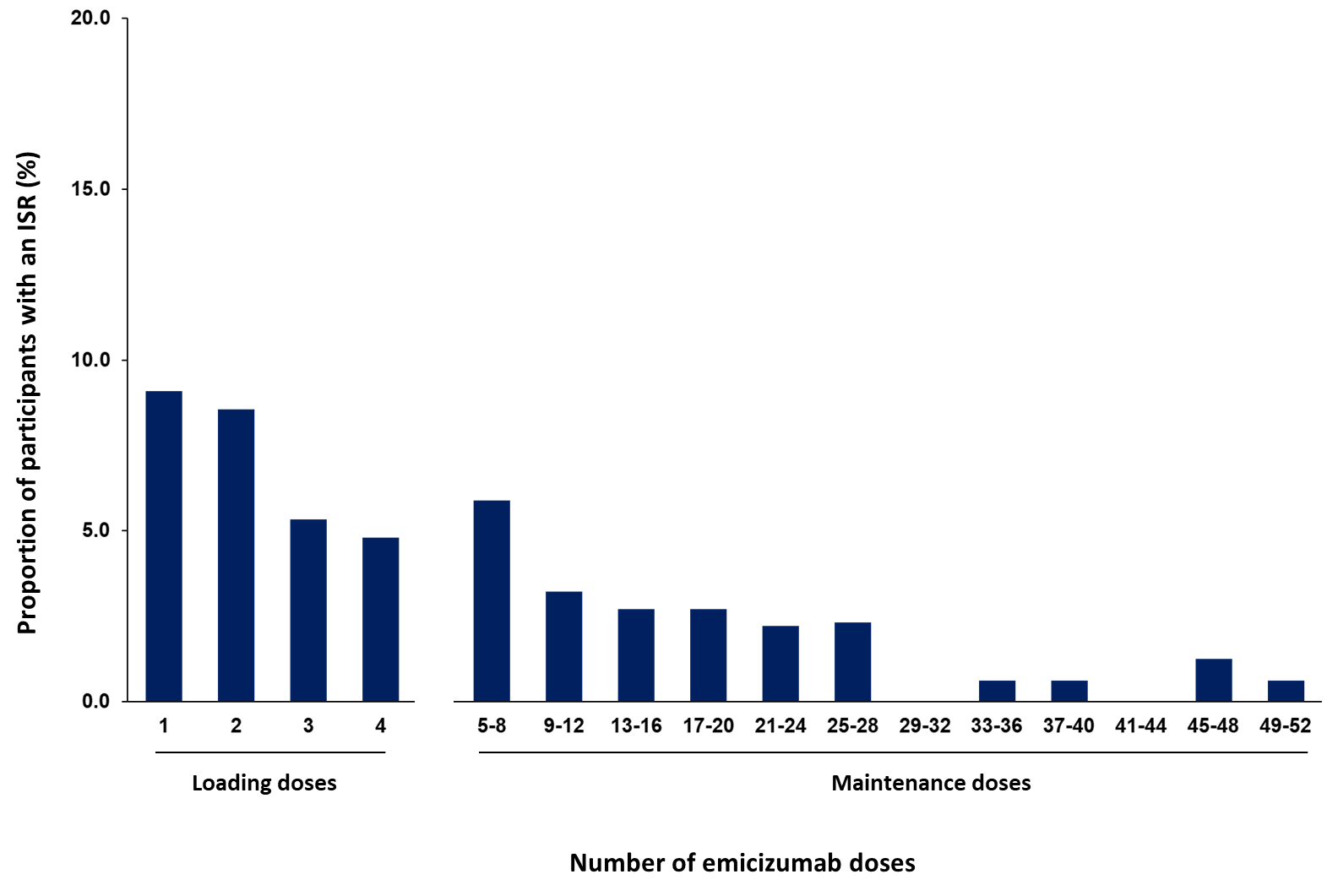

Supplement: Supplementary Tables and Figures [file mmc1.docx]
